# Effects of Monochromatic Illumination with LEDs Lights on the Growth and Photosynthetic Performance of *Auxenochlorella protothecoides* in Photo- and Mixotrophic Conditions

**DOI:** 10.3390/plants10040799

**Published:** 2021-04-19

**Authors:** Giorgos Markou, Alexandros Diamantis, Evagelina Korozi, Vasiliki Tsagou, Io Kefalogianni, Iordanis Chatzipavlidis

**Affiliations:** 1 Institute of Technology of Agricultural Products, Hellenic Agricultural Organization-Demeter, L. Sof. Venizelou 1, 14123 Lykovrysi, Greece; diamalexandros92@gmail.com; 2 Laboratory of General and Agricultural Microbiology, Department of Crop Science, Agricultural University of Athens, Iera Odos 75, 11855 Athens, Greece; evangeliakorozi@yahoo.com (E.K.); tsagouv@hotmail.com (V.T.); bmic7kei@aua.gr (I.K.); chatzipavlidis@aua.gr (I.C.)

**Keywords:** mixotrophy, glycerol, monochromatic illumination, photosynthetic performance, microalgae, single-cell protein

## Abstract

This study examined the effects of monochromatic illumination (blue, red, green and yellow) employing light-emitting diodes (LEDs), trophic conditions (photoautotrophic and mixotrophic), and nitrogen availability (high and low peptone concentration) on the growth and biochemical composition of *Auxenochlorella protothecoides*. The results revealed that mixotrophic conditions did not favor *A. protothecoides*, giving lower growth rates compared to heterotrophy (dark conditions). However, mixotrophy gave significantly higher growth rates compared to photoautotrophy. The best light wavelengths for mixotrophic cultivation were that of white and red. In all cases investigated in this study, high peptone concentration (4 g/L) resulted in decreased growth rates. Regarding the biochemical composition of *A. protothecoides*, the strongest effect, irrespective of trophic conditions, was caused by nitrogen availability (peptone concentration). Specifically, at nitrogen replete conditions (4 g/L peptone), biomass was rich in proteins (32–67%), whereas under deplete conditions (0.5 g/L peptone), *A. protothecoides* accumulated mainly carbohydrates (up to 56%). Mixotrophic conditions generally favored higher carbohydrate content, whereas photoautotrophic conditions favored higher protein content. The different illumination spectra did not have any clear effect on the biochemical composition (metabolites content), except that, in all trophic conditions, the use of the green spectrum resulted in higher chlorophyll b content. Chlorophyll a fluorescence studies revealed that the trophic conditions and the high peptone concentrations impacted the photosystem II (PSII) performance, and also affected plastoquinone re-oxidation kinetics and the heterogeneity of the PSII reaction centers.

## 1. Introduction

Microalgae are an important renewable source for the production of various biomolecules applied in different sectors (food industry, chemical industry, medicine, etc.) and offer numerous possibilities for developing a modern bioeconomy [1]. Microalgae are mainly photoautotrophic (photosynthetic) microorganisms and thus have attracted interest as a means of harvesting light energy and converting it into valuable metabolites [1,2]. However, microalgae display flexibility in their metabolism and are capable of growing under different trophic conditions, namely photoautotrophy, heterotrophy, and mixotrophy. Under photoautotrophic growth conditions, microalgae utilize sunlight as an energy source and fix CO_2_. Under heterotrophic conditions, microalgae utilize organic molecules, such as glucose, glycerol, and acetate, as energy and carbon sources, and under mixotrophic conditions, in addition to the effects of heterotrophy, they also perform photosynthesis and can simultaneously utilize CO_2_ and organic substrates as a carbon source [3]. Mixotrophic growth conditions are of particular importance, particularly at the industrial scale, because they overcome the light limitation of photoautotrophy and significantly reduce the requirements of large land areas for the production of microalgal biomass [4]. A suitable organic carbon source for the mixotrophic or heterotrophic growth of microalgae is glycerol, a waste-stream of industrial biodiesel production that is available in large quantities [5]. Α highly promising microalgal species for glycerol utilization is *Auxenochlorella protothecoides*, and several studies have demonstrated its strong potential to be grown using glycerol [6,7,8,9,10].

Light intensity (quantity) and light spectra (quality) are important parameters for microalgal growth. Although microalgal growth using solar energy is the most economical way to produce microalgae, it requires large outdoor areas and also suffers from the eventually low photosynthetic efficiency observed at full sunlight intensities, i.e., the low conversion of light energy into biomass (in practice 1–1.5% of solar light) equivalent to 2–3% of photosynthetically active radiation. Even at the maximum theoretical efficiency of solar energy conversion (≈11%), the strongest illuminated outer cell layers lead to a high dissipation rate (>80%) of light energy because unused thermal energy activates photoprotective mechanisms (such as non-photochemical quenching), thus resulting in overall low photosynthetic efficiencies. This high energy dissipation is due to the mismatch between the absorption and the utilization of energy during the dark reactions [11,12,13]. Artificial illumination can significantly increase photosynthetic efficiency by avoiding photoinhibition due to high light intensities [14]. Light spectra have an important impact on the photosynthetic process and hence on growth because not all light wavelengths are absorbed equally by the photosynthetically active pigments of microalgae. Chlorophyll *a* is the core photosynthetically active pigment of the reaction centers, whereas chlorophyll *b*, *c* and *d* are accessory pigments that extend the range of light absorption. Chlorophylls (green pigments) have two major absorption bands: blue or blue-green (450–475 nm) and red (630–675 nm) [15]. Moreover, several studies have demonstrated that the light quality has an impact on the biochemical composition of microalgae, triggering the accumulation of major metabolites, such as lipids, proteins, and carbohydrates, or pigments such as chlorophylls, carotenoids, or cyanobacterial phycocyanin [16,17,18,19]. Light-emitting diodes (LEDs) can serve as an energy efficient light source for microalgal growth compared to traditional lighting (halogen bulbs, fluorescence bulbs, incandescent bulbs, etc.), because they emit only at given bands of wavelengths that are absorbed by the photosynthetic pigments [18,20]. Moreover, LEDs can potentially serve as an effective light source providing improved capabilities for the control and manipulation of the biochemical composition of microalgae. However, there is a lack of published research work using monochromatic illumination for the growth and the manipulation of the biochemical composition of microalgae grown under mixotrophic conditions.

Chlorophyll fluorescence analysis is a highly useful tool for obtaining information about the photosynthetic process and in particular of the photosystem II (PSII). Although a vast literature exists on chlorophyll fluorescence studies on higher plants and photosynthetic microorganisms (such as microalgae) grown under different conditions (focusing mainly on stress conditions), few data are available regarding chlorophyll fluorescence of mixotrophically grown microalgae [21,22]. Hence, the overall aim of this work was to study the effect of selected monochromatic LEDs (blue, green, yellow, and red) on the growth and biochemical composition of the microalga *A. protothecoides*. In addition, this study aimed to study the effect of the trophic conditions on the PSII performance through chlorophyll fluorescence measurements and analysis.

## 2. Results and Discussion

There is an increased interest in utilizing artificial light for growing microalgae, particularly for the production of high value products, such as pigments, unsaturated fatty acids, and proteins. Among different artificial light types, the use of LEDs is considered to be the most energy efficient, mainly because they emit precise light wavelength bands that can be absorbed by the photosynthetic antenna [23,24]. Therefore, in this study different LED colors were used to assess the growth ability of *A. protothecoides* and its biochemical composition changes in photoautotrophic and mixotrophic metabolisms. The cultures with low glycerol concentration (0.5 g/L) were considered as photoautotrophic, and those with high glycerol concentration (10 g/L) as mixotrophic (see Section 3.2 for more details on the experimental design).

### 2.1. Effect of Trophic Conditions and Monochromatic Illumination on the Growth Rates of A. protothecoides

#### 2.1.1. Photoautotrophic Conditions

**Figure 1:**
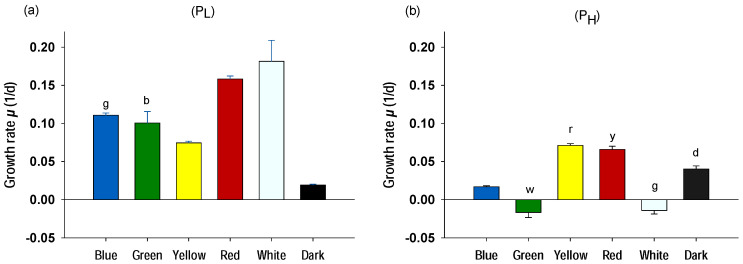
Growth rates of *A. protothecoides* cultivated photoautotrophically for seven days with different light wavelengths: (**a**) with low peptone (P_L_) and (**b**) with high peptone (P_H_). Each bar represents the average ± SD of n = 3 replicates. The letters (b: blue, g: green, y: yellow, r: red. w: white, and d: dark) indicate that there were no statistically significant differences between the means (two-way ANOVA; Duncan method; *p* < 0.05) of the pairwise comparisons between the different wavelengths. Between the variable groups P_L_ and P_H_ there were statistically significant differences (*p* < 0.01).

Figure 1 presents the effect of the trophic conditions and the monochromatic illumination on the growth rates of *A. protothecoides* with 0.5 and 4 g/L peptone (P_L_ and P_H_, respectively). Figure 1a shows the photoautotrophic cultures with low peptone concentration (P_L_), which are considered to reflect a typical form of photoautotrophy (see Section 2.2 for more details). The highest growth rates were obtained with white LEDs (0.18 1/d) followed by red (0.158 1/d; with statistically significant differences between them; *p* < 0.05), whereas blue (0.111 1/d), green (0.101 1/d), and yellow light (0.074 1/d) displayed much lower growth rates. However, all cultures illuminated with monochromatic lights had significantly higher growth rates compared to the negative control (grown on dark conditions). Because the P_L_ cultures grew photoautotrophically, the differences in the growth rates reflect the diverse ability of *A. protothecoides* to harvest the different light wavelengths. Figure A2 (see Appendix A) shows the spectrum analysis of each monochromatic LED used in the study and the associated absorption peaks of the different pigments of the photosynthetic antenna of microalgae. Based on the absorption peaks of chlorophyll a, b, and carotenoids, yellow and green lights are poorly absorbed, whereas white, blue, and red are absorbed by the main photosynthetic pigments. Because white, red, and blue are the main light wavelengths that are absorbed by the photosynthetic pigments, it was initially hypothesized that they would display higher growth rates compared to the poorly absorbed green and yellow. The effects on the P_L_ cultures confirm this hypothesis. A few other reports confirm that microalgae do not grow well under green light [25,26,27] because this wavelength is poorly absorbed by the photosynthetic antenna and cannot provide useful energy for photosynthesis. However, there is disagreement concerning yellow light, as Hultberg et al. [26] and de Mooij et al. [28] found that yellow light was very effective for biomass production of *Chlorella vulgaris* and *Chlamydomonas reinhardtii*, respectively. de Mooij et al. [28] indicate that yellow light could be used more efficiently, especially when light is provided in high densities compared to red, blue, or white because the latter result in an oversaturation of the photosynthetic antenna, whereas the excess light is wasted as energy through heat dissipation. However, because light was provided in the present study significantly below the saturation point (usually around 200–400 μmol/m^2^/s) [15], yellow light resulted in lower growth rates compared to red, white, and blue.

In Figure 1b the effect of the different light wavelengths on the cultures with high peptone (P_H_) content is shown. In all cases there is a marked decrease (*p* < 0.01) in the growth rates compared to the low peptone cultures (P_L_), except for the negative control (dark conditions), where it seems that peptone acted in the long term as an energy source for cells and supported a slightly better growth (see Section 3.2 for more details on peptone used as a carbon source). The decreased growth rates of P_H_ suggest that peptone at relative higher concentrations has an inhibitory effect on *A. protothecoides* grown under photoautotrophic conditions. Among the wavelengths that are more strongly absorbed by the photosynthetic pigments, blue LEDs showed significantly lower growth rates compared to white and red. These results are in agreement with the study of Chen and Su [29], who also obtained lower biomass production using blue light in *Auxenochlorella pyrenoidosa* compared to red and white. Blue light has shorter wavelengths and therefore contains higher energy [30]. Hence, it is more likely to cause photo-inhibition. However, various studies demonstrate that blue color is very effective and promotes higher biomass production in other microalgal species [19,24,31]. The diverse results on the literature reflect that the effectiveness of light wavelength on biomass production could be species dependent. This could be the outcome of the content and ratio of photosynthetically active pigments, the light saturation level, and in general the physiological characteristics of each microalgal species [15,32]. The present results suggest that *A. protothecoides* reproduction capacity is not favored by the illumination with blue light.

#### 2.1.2. Mixotrophic Conditions

**Figure 2:**
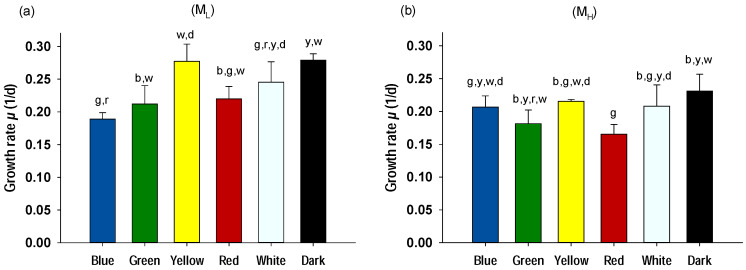
Growth rates of *A. protothecoides* cultivated mixotrophically for seven days with different light wavelengths: (**a**) with low peptone (M_L_) and (**b**) with high peptone (M_H_). Each bar represents the average ± SD of n = 3 replicates. The letters (b: blue, g: green, y: yellow, r: red. w: white, and d: dark) indicate that there were no statistically significant differences between the means (two-way ANOVA; Duncan method; *p* < 0.05) of the pairwise comparisons between the different wavelengths. Between the variable groups M_L_ and M_H_ there were statistically significant differences (*p* < 0.01).

Figure 2 illustrates the growth rates of *A. protothecoides* grown mixotrophically on glycerol with 0.5 and 4 g/L peptone (M_L_ and M_H_, respectively) and illuminated with different light wavelengths. Mixotrophy gave significantly higher growth rates (*p* < 0.01) compared to the autotrophic conditions, which shows the positive response and ability of *A. protothecoides* to grow on glycerol as a source of energy and organic carbon. Dark conditions in the presence of glycerol (heterotrophic) gave the highest growth rates for both M_H_ and M_L_ (0.231 and 0.279 1/d, respectively). In the series of cultures with low peptone concentration (M_L_; 0.5 g/L), the highest growth rates were obtained under mixotrophic conditions with yellow illumination (0.277 1/d), followed by white illumination (0.245 1/d), red and green (0.220 and 0.212 1/d, respectively), whereas blue light displayed the lowest values (0.189 1/d). As in photoauthotrophic conditions, under mixotrophy the increased peptone concentration also showed lower growth rates in all individual treatments studied (*p* < 0.01). Among the light wavelengths tested on M_H_ cultures, yellow, white, and blue gave the highest values (0.215, 0.208, and 0.207 1/d, respectively) followed by green and red lights (0.181 and 0.165 1/d, respectively).

The lower growth rates of illuminated cells were most probably due to a potential inhibition effect of light on *A. protothecoides* grown mixotrophycally. As was reported by Xiao et al. [33], the proteomics analysis of *A. protothecoides* cultivated in glucose revealed that light had an apparent restrictive effect on the metabolic process of organic carbon assimilation. In addition, it was demonstrated here that the mixotrophic conditions also affected the photosynthetic process and the photosynthetic apparatus heterogeneity of *A. protothecoides*. The various parameters and indexes of chlorophyll fluorescence (such as F_v_/F_m_ and *PI_ABS_*) revealed that in the presence of glycerol the overall photosynthetic performance was reduced (see Section 2.3). These results suggest that *A. protothecoides* is not favored by the mixotrophic conditions, probably due to a combination of reduced organic carbon assimilation and lower photosynthetic performance. It should be noted however that various microalgal species, for example *Nannochloropsis* sp. (Xu et al., 2004) or *Platymonas subcordiformis* (Xie et al., 2001), display higher growth rates and grow more quickly under mixotrophic conditions and therefore mixotrophy is considered as a beneficial culturing technique [34]. Α three-way ANOVA analysis revealed that there was a statistically significant difference (*p* < 0.01) between all three variable categories studied (light wavelengths, glycerol presence, and high/low peptone concentrations), confirming that each one of the three variables is a significant bioprocess parameter that influences the overall growth capability of *A. protothecoides*.

### 2.2. Biochemical Composition and Pigment Content of A. protothecoides

**Figure 3:**
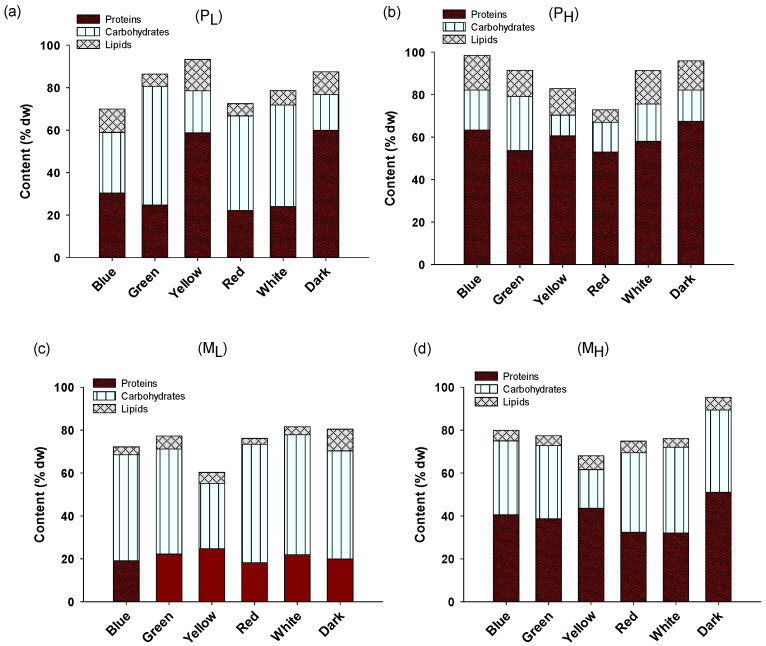
Biochemical composition of *A. protothecoides* cultivated in different light wavelengths under photoautotrophic conditions with (**a**) low peptone and (**b**) high peptone concentration, and in mixotrophic conditions with (**c**) low peptone and (**d**) high peptone concentration. Each bar represents the average of n = 3 replicates.

Figure 3 illustrates the biochemical composition (proteins, lipids, and carbohydrates) of *A. protothecoides* cultivated in the different conditions tested in this study. The main trend observed was that cultures with higher peptone concentrations (P_H_ and M_H_) generally displayed increased (*p* < 0.01) protein content (49.5% on average) compared to P_L_ and M_L_ (29% on average) apparently due to the higher availability of nitrogen. Carbohydrate content was in general higher (*p* < 0.01) in the associated cultures with lower peptone content (42.5% vs. 29% on average in low and high peptone, respectively). Between the trophic conditions, photoautotrophic cultures gave higher contents for protein (48% on average) and lipid (11% on average), and lower for carbohydrates (26% on average) compared to the mixotrophic (30%, 5%, and 41% on average for proteins, lipids, and carbohydrates, respectively) (*p* < 0.01). Regarding lipid content, it was found that higher availability of nitrogen in the photoautotrophic cultures resulted in higher lipid content (*p* < 0.05). These results are not in line with other studies on *A. protothecoides*, which report lipid contents of more than 50% [35,36]. It appears that the strain used in this study under the specific conditions resulted in the accumulation either of proteins (when grown under nitrogen replete conditions) or carbohydrates (when grown under low nitrogen availability). It is well documented that under nitrogen limitation, microalgae change their metabolic pathways towards the synthesis of carbonaceous compounds (carbohydrates or lipids). In particular, the accumulation of carbohydrates has been observed as the first response of microalgae to nitrogen starvation, whereas lipids start to accumulate in later cultivation stages [37].

Regarding the effects of light wavelength, yellow light and dark conditions displayed the highest protein content, whereas the wavelengths white, red, and blue resulted in the triggering of the accumulation of carbohydrates. Overall, the three-way ANOVA analysis revealed that there was a significant interaction between the two variables (peptone concentration and trophic conditions), whereas the light wavelength did not result in statistically significant interactions. These results suggest that biochemical composition was ruled mainly by the availability of nitrogen and the trophic condition. Regarding the effect of light wavelength on lipid production, no clear observation was drawn (no statistically significant differences *p* > 0.05). Overall, there was a strong negative correlation (R^2^ = 0.83) between the protein and carbohydrate content, which indicates that the accumulation of one of them was accompanied by the decrease in the content of the other. No other strong correlation between proteins or carbohydrates and lipids or pigments was obtained (R^2^ < 0.5).

**Figure 4:**
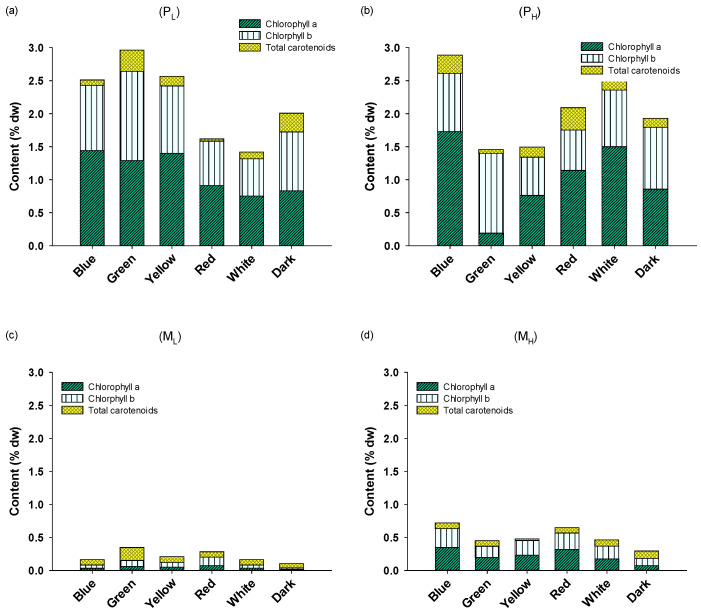
Pigments content of *A. protothecoides* cultivated in different light wavelengths under photoautotrophic conditions with (**a**) low peptone and (**b**) high peptone concentration, and in mixotrophic conditions with (**c**) low peptone and (**d**) high peptone concentration. Each bar represents the average of n = 3 replicates.

Mixotrophy significantly affected the pigment content (*p* < 0.01) (Figure 4), which was highly decreased (from about 2.1% on average for all pigments for P_L_ and P_H_ to about 0.35% on average for M_L_ and M_H_). It has been frequently reported that mixotrophy itself influences the pigment content of microalgae [38], because in the presence of organic molecules (such as glucose or glycerol) cells synthesize less photosynthetically active pigments. Thus, under mixotrophic conditions cells can harvest energy from the organic compounds and are therefore less dependent on light availability compared to the photoautotrophic conditions, under which they absolutely depend on light energy for their growth [38]. A strong effect was also observed in green light, where chlorophyll b content was the highest in both P_L_ and P_H_ cultures (1.35% and 1.21%, respectively). No other clear effect of light wavelength on pigments was observed. Three-way ANOVA analysis revealed that the only statistically significant variable was the trophic conditions, and any other interaction was not significant.

### 2.3. Chlorophyll Fluorescence Studies

#### 2.3.1. Maximum Quantum Yields of Primary Photochemistry and Non-Photochemical Quenching (NPQ)

**Figure 5:**
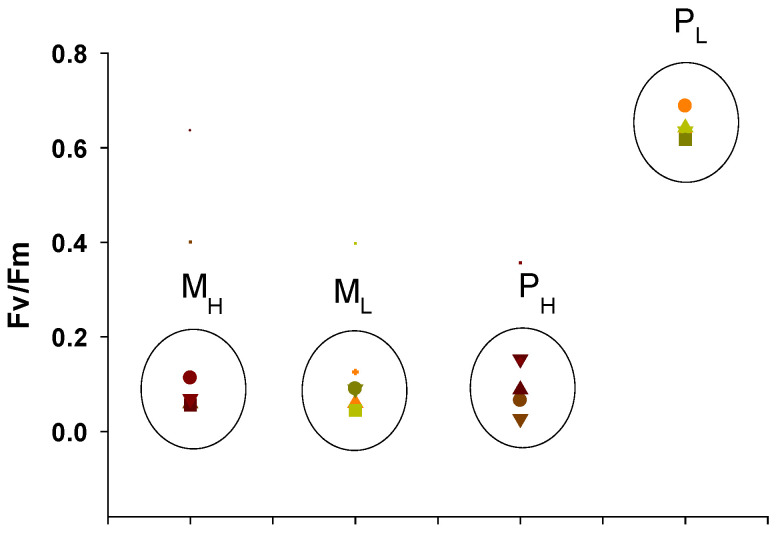
Maximum quantum yields of primary photochemistry (F_v_/F_m_) of *A. protothecoides* cultivated under the different conditions (each circle represents a different cultivation treatment; M_H_ and M_L_ stand for mixotrophy with high and low peptone, respectively, and P_H_ and P_L_ stand for photoautotrophy with high and low peptone, respectively).

Figure 5 illustrates the maximum quantum yields for primary photochemistry (F_v_/F_m_) of *A. protothecoides* cultivated under the different conditions. The results clearly show that the P_L_ cultures, which represent the most typical photoautotrophic conditions, displayed F_v_/F_m_ values in the range of 0.62–0.69, while all other cultures (M_H_, M_L_, and P_H_) had F_v_/F_m_ in the range of 0.05–0.15. These results indicate that in the presence of organic substrate (glycerol) and under relatively high concentration of peptone, the photosynthetic efficiency of *A. protothecoides* was impacted and all the calculated parameters of the chlorophyll fluorescence analysis (Figure 5, Table 1) were significantly different compared to the P_L_ cultures. The overall results (data not shown) indicated that there were similar trends in the chlorophyll fluorescence analysis of *A. protothecoides* grown under the different wavelengths, suggesting that the main factors influencing the photosynthetic performance were the trophic conditions and the availability of nitrogen. Therefore, in the following, for an easier discussion of the findings, only the results of the white LEDs as the reference light source are shown.

Table 1 lists the main calculated OJIP and non-photochemical quenching (NPQ) parameters that give an overview of the photosynthetic performance and photoprotection process. As shown in the inset figures of Table 1**,** chlorophyll fluorescence kinetics strongly impacted the cultures M_H_, M_L_, and P_H_. The presence of glycerol in M_H_ and M_L_, i.e., the switching of the trophic conditions into mixotrophy and probably the alteration of the pigment content (Section 3.2) strongly impacted the photosynthetic processes, as shown by the chlorophyll fluorescence analysis. Regarding P_H_, the decrease in F_v_/F_m_ should be related to some kind of inhibitory effect of peptone, because it is also reflected by the impacted growth rates (see Section 2.3.1). The most apparent changes revealed by the OJIP test were the increased absorption of light per reaction center (RC) and the increased dissipated energy (DI_o_/RC and *φ*_Do_) of *A. protothecoides* in all three series (M_H_, M_L_, and P_H_). This subsequently had a strong effect on PI_ABS_ (Table 1). The parameter PI_ABS_ is an index that reflects the functionality of PSII, giving quantitative information on the current state of the PSII performance. This index can indicate whether the photosynthetic microorganisms are under stress conditions or whether they diversify their photosynthetic process for other reasons [39].

NPQ and *qp* values were very low for M_H_ and P_H_, whereas they were much higher for M_L_ and P_L_. These results suggest that under high peptone concentration the lower values of NPQ and *qp* indicate that non-photochemical and photochemical mechanism(s) were not able to be activated, and thus might cause photoinhibition, leading subsequently to lower growth rates. The low values of NPQ and *qp* may indicate possible alterations in the proton gradient formation across the thylakoid membrane. In contrast, M_L_ and P_L_ had significantly higher NPQ values, and therefore in these cultures the excess of light energy was probably dissipated more efficiently. These findings are more profound considering that growth rates were significantly and negatively impacted in P_H_. This fact suggests that mixotrophic conditions could provide somehow a growth protection against photoinhibition. Li et al. [40] observed that in the cyanobacterium *Arthrospira platensis* the addition of glucose protected against strong photoinhibition caused by ammonia toxicity. This point deserves to be more thoroughly investigated.

#### 2.3.2. Q_A_^−^ Re-Oxidation Kinetics on Cultures with White Light

Table 2 shows the half-time (*t*_1/2_) of the three phases of the Q_A_ re-oxidation kinetics (inset figures) without and with 50 μM 3-(3,4-dichlorophenyl)-1,1-dimethylurea (DCMU). In the kinetics without DCMU, the fast phase, which is related to the re-oxidation of Q_A_^−^ by Q_B_, was significantly affected by the trophic conditions and the peptone concentration, while the medium phase was not altered for M_H_ and P_L_ and was much slower for M_L_ and P_H_. The slow phase had higher values in M_H_ and P_H_ than in P_L_. In the presence of DCMU, M_H_, M_L_, and P_H_ displayed faster half times compared to P_L_, whereas the medium and slow phases were significantly slower (Table 2). The medium and slow phases reflect the binding of quinone to the Q_B_ site and the recombination of Q_A_^−^ with the S_2_ state of the oxygen evolving complex (OEC), respectively [41,42]. The faster kinetics of Q_A_ re-oxidation in P_L_ suggest a faster re-opening of RCs, which is consistent with the higher *qp* values also found in this culture. The Q_A_^−^ re-oxidization depends mainly on the rate of forward electron transport and consequently on the redox state of the electron transfer chain, whereas in the presence of DCMU, the Q_A_^−^ re-oxidation kinetics are an indicator of the condition of the donor side of PSII [42]. Overall, these results suggest that the trophic conditions and the peptone concentration have a strong effect on the electron transfer chain process. This could be an outcome of the overall alteration of some metabolic pathways, and of cell organization under the mixotrophic conditions, as was also shown for *Chlorella vulgaris* after transcriptomics and proteomics analysis [43].

#### 2.3.3. Inactive PSII RCs and Q_B_ Non-Reducing RCs on Cultures with White Light

The S-States test, which was conducted for the determination of the inactive PSII reaction centers (Table 3), revealed that the only involved inactive PSII RC was observed in P_L_ cultures, where 8.5% of the total PSII RCs was inactive. In the other cultures, the figures were negative values, suggesting that under mixotrophic conditions or high peptone concentration no inactive PSII RCs were present because no fluorescence decay after the fourth flash (S_4_ → S_0_/S_1_) was observed. However, it should be noted that the fluorescence signals in M_L_ were significantly weaker than in the other cultures and that possibly the calculations were influenced by strong noise. Moreover, the double OJIP protocol revealed that the Q_B_ non-reducing RCs were almost doubled in the M_L_, M_H_, and P_H_ compared to the P_L_ (Table 3). These results suggest that the trophic conditions and the peptone concentration have a strong effect on the heterogeneity of the PSII reaction centers. However, no further literature is available on this topic and more research is needed.

## 3. Materials and Methods

### 3.1. Microorganism and Cultivation Conditions

*Auxenochlorella protothecoides* (CCAP 211/8D) was taken from the Culture Collection of Algae and Protozoa SAMS Limited Scottish Marine Institute. The basic growth medium (BGM) consisted of 0.5 g/L KH_2_PO_4_, 25 mg/L CaCl_2_, 10 mg/L Na_2_EDTA, 75 mg/L MgSO_4_·7H_2_O, 5 mg/L FeSO_4_·7H_2_O and 1.0 mL of trace elements stock solution: 2.86 g/L H_3_BO_3_, 20 mg/L (NH_4_)6Mo_7_O_24_, 1.8 g/L MnCl_2_·4H_2_O, 80 mg/L CuSO_4_·5H_2_O and 220 mg/L ZnSO_4_·7H_2_O. For the growth of stock cultures, 2 g/L peptone as nitrogen source and 5 g/L glycerol as organic carbon source were used. The inoculum cultures, which were used to inoculate the main experimental cultures, were grown with 2.5 g/L glycerol for three days under cool white LED panel illumination (100 μmol/m^2^/s; measured on the top of the transparent basis on which the photobioreactors where placed, i.e., on the outside of the bottom of the photobioreactors. Photon flux was measured with a SpectraPen LM 510 (Photon Systems Instruments, Czech Republic)). The experimental cultures were inoculated with 20% of inoculum cultures.

**Figure A1:**
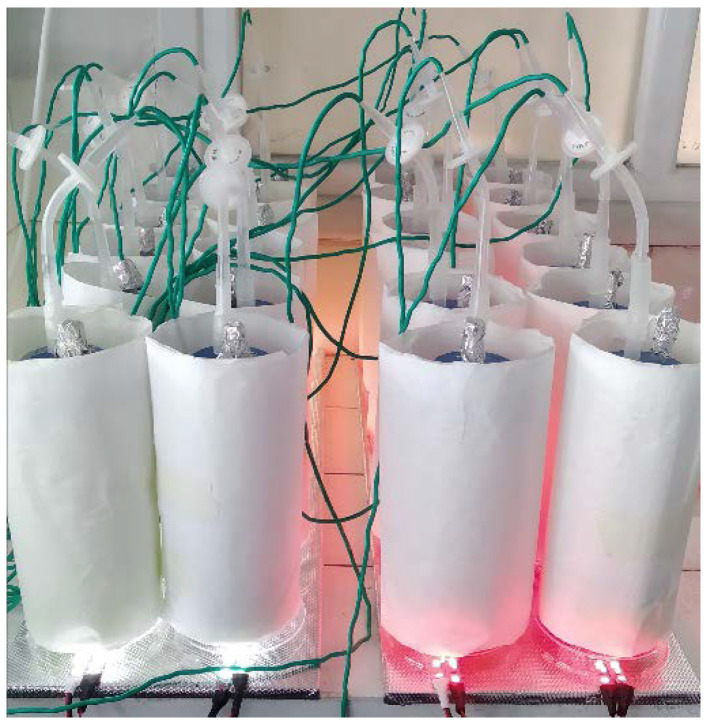
Experimental configuration for growing *A. protethecoides* with monochromatic illumination by LEDs in 500 mL flasks aerated with sterilized air.

**Figure A2:**
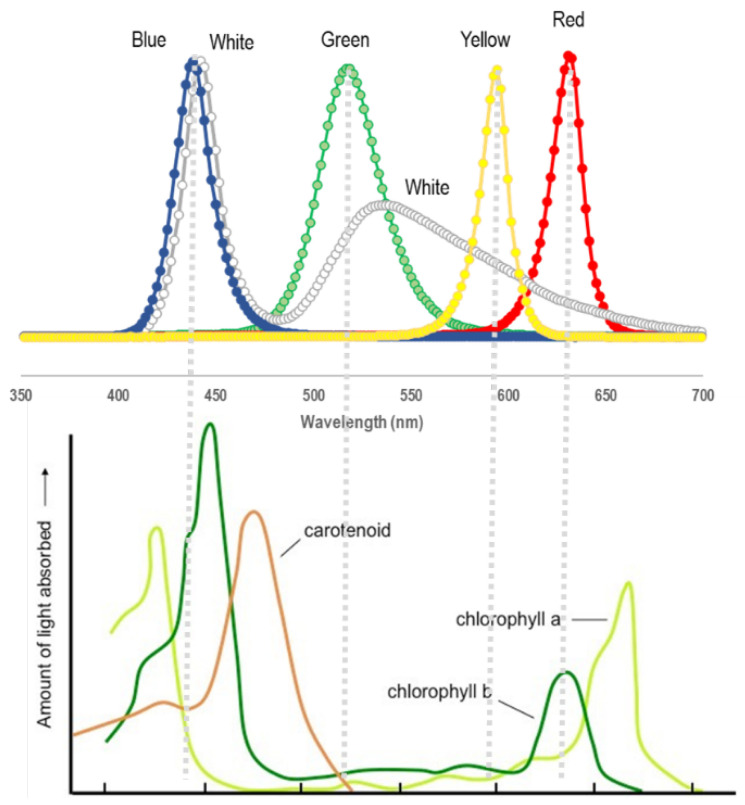
Spectra of the LEDs used for the illumination of the cultures of *A. protothecoides* and the associated absorption of the photosynthetically active pigments (chlorophyll *a*, chlorophyll *b*, and carotenoids).

All cultivations were performed in 500 mL Duran flasks with a working volume of 300 mL (250 mL of fresh growth medium and 50 mL of inoculum). All cultures were axenic and performed under sterile conditions. The cultures were aerated with 0.2 μm filter-sterilized air for agitation and oxygen enrichment. Illumination was provided by five LEDs (SMD type; 14.4 W per meter, 60 SMD LEDs per meter; GloboStar, Greece) per flask (Figure A1 in the Appendix A). Illumination was provided from the bottom of the flasks at a photon flux of 100 ± 5 μmol/m^2^/s. Four monochromatic colors of illumination were used, namely blue, red, green, and yellow. Cool white LEDs were employed as controls (standard illumination). The Spectrum analysis of each monochromatic LED is shown in Figure A2 in the Appendix A.

### 3.2. Experimental Design

For each different LED light, four cultures were used (Table 4), two in which *A. protothecoides* was grown mixotrophically on glycerol (10 g/L) (M_H_ and M_L_ = mixotrophic conditions (M) with high (H) or low (L) peptone concentration)–and two cultures of *A. protothecoides* grown photoautotrophically (P_H_ and P_L_ = photoautotrophic conditions (P) with high (H) or low (L) peptone concentration), applying also KHCO_3_ as an inorganic carbon source. For mixotrophic and photoautotrophic cultures, the photoperiod was set at 16 h light and 8 h dark. The overall duration of the cultures was 7 days. The varied nitrogen (peptone) concentrations aimed, in addition to the light wavelength, at investigating the effect of nitrogen availability on the growth, biochemical profile, and photosynthetic efficiency of *A. protothecoides*. Moreover, in preliminary trials it was obtained that *A. protothecoides* is unable to uptake nitrates or ammoniacal nitrogen as a nitrogen source, therefore the use of peptone as a nitrogen source was also unavoidable in the photoautotrophic cultures. However, because peptone is an organic compound that probably can also be used as organic carbon, the low peptone concentration (0.5 g/L) in the P_L_ cultures aimed at ensuring that at the time point of conducting the Chl-fluorescence analyses on day 2, *A. protothecoides* had consumed all the available peptone and therefore grew under photoautotrophic conditions. Thus, P_L_ cultures were considered as the main representative of the photoautotrophic conditions in this study, whereas P_H_ due to the higher peptone concentration could not be fully considered as photoautotrophic cultures. However, for the sake of the simplicity of the results presentation, photoautotrophic conditions were assessed in both P_L_ and P_H_. A series of cultures kept in the dark for the same duration as the illuminated ones were performed in order to see if the microalga could grow heterotrophically using as sole carbon and energy sourcethe organic substances provided (glycerol and peptone). Dark grown cultures are referred to as negative controls.

### 3.3. Analytical Methods

#### 3.3.1. Biomass Analysis

Biomass production as dry weight was measured after separating cells from the growth medium through centrifugation (2500 rpm for 10 min) and washing them with deionized (DI) water at least 3 times to remove and exclude the culture medium, and drying overnight in an oven (80 °C) until constant weight. Biomass concentration was also measured indirectly by the optical density (OD) at the wavelength of 750 nm [44]. Specific growth rates (μ) were calculated as: μ = (lnN1 − lnxN2)/(t), where N1, and N2 are the biomass concentrations after the inoculation of the cultures (N1) and the time of harvesting (N2), respectively after 7 days of cultivation (t).

Carbohydrates were measured by a modified phenol-sulfuric acid method [45]: briefly, in 0.5 mL of cell sample containing 10–50 mg/L carbohydrates, 10 μL of 90% phenol solution were added and mixed, followed by the addition of 1.25 mL of concentrated sulfuric acid (96%). After 30 min OD was measured at 485 nm using D-glucose as the standard sugar. Lipids were measured by a modification of the sulfo-vanillin method [46] after extraction of lipids with 2:1:0.2 chloroform:methanol and water. Briefly, 20 μL of extracted sample containing 200–500 mg/L of lipids were incubated in 80 °C to evaporate chloroform and then 0.4 mL of 96% sulfuric acid w added and samples were placed in boiling water for 10 min. Samples were then left at room temperature for 15 min to cool and 1.0 mL of phosphoric-acid/vanillin solution was added (solution stock was prepared by dissolving 0.12 g of vanillin in 20 mL DI water and finally in 80 mL of 85% phosphoric acid). The samples were incubated at 37 °C for 15 min and OD was measured at 530 nm. For the standard curve maize oil was used. Proteins were assayed according to Lowry et al. [47] after the extraction with 0.5 N NaOH: in brief, 1.5 mL of samples were centrifuged, the pelleted biomass was resuspended in 1.5 mL 0.5 N NaOH and then incubated on an agitation heating plate at 100 °C for 20 min. An aliquot of 100 μL of extracted proteins was then added to 100 μL 5% SDS, and supplemented with 1 mL of a solution consisting of 2% Na_2_CO_3_ in 0.1 N NaOH. After 15 min, 100 μL of freshly prepared 1 N Folin and Ciocalteu reagent was added and samples were left for 30 min in the dark. OD was measured at 750 nm using bovine serum albumin as the standard. Chlorophylls and total carotenoids were extracted with 90% methanol. In short, 2 mL of samples were centrifuged and the pellet was suspended in 2 mL of 90% methanol and incubated at 70 °C for 5 min. The concentrations of chlorophylls and total carotenoids were measured according to the equations given by Lichtenthaler [48]. All biomass composition analyses were performed after the samples were washed several times with DI water. All spectrophotometric determinations were carried out by a Cadas 50 (Dr. Lange GmBH, Saarbrücken, Germany) spectrophotometer and analyses were carried out at least in triplicate for each replicate. The final data given are the average of three analytical replicates from each of the three biological replicates (n = 3) with standard deviation. Statistical analysis was based on analysis of variance ANOVA (one-, two-, and three-way comparisons), conducted using SigmaPlot 12.0 software (Systat Software, Inc., San Jose, CA, USA). All data were tested for Normality (Shapiro–Wilk test) and for equal variance between treatments. Post-hoc statistical analysis was based on Duncan’s pairwise multiple comparison procedure.

#### 3.3.2. Chlorophyll Fluorescence Measurements and Analysis

Chlorophyll fluorescence measurements were taken after the adaptation of cells in the dark (>20 min). The fluorescence transients of the OJIP test reflect the kinetics during the reduction of the plastoquinone (PQ) pool [14]. The model of Q_A_ on which the chlorophyll fluorescence analysis is based can be described briefly as follows: photons are absorbed by the antennae pigments (absorption flux: ABS) which are excited. A part of the excitation energy, the trapping flux (TR), is transferred to the reaction center (RC) while the remaining part is dissipated either as fluorescence or as heat. The TR is converted in the RCs to redox energy by reducing the electron acceptor Q_A_ to Q_A_^−^, which is then re-oxidized to Q_A_ leading to electron transport (ET) and consequently to photochemistry [39]. The terms and formulae for the various parameters of the OJIP test are shown in Table 5 and were based on Strasser et al. [39] unless otherwise stated in the text.

**Photochemical and non-photochemical quenching:** Photochemical and non-photochemical quenching measurements and analysis were routinely performed using an AquaPen 110-C fluorometer (PSI Instruments, Czech Republic). The NPQ protocol begins by measuring the minimal level of fluorescence F_o_ in dark-adapted cells. After recording F_o_, a short saturating flash reduces the PQ pool and F_m_ is recorded. A short dark relaxation period is followed, and then actinic light is supplied for tens to hundreds of seconds to provoke the Kautsky effect and fluorescence signals (F_t_) are recorded. Then, a sequence of additional saturating flashes is applied on top of the actinic light to measure the NPQ and the effective quantum yield measuring F_m_’ in light adapted state. NPQ was calculated as (F_m_ − F_m_’)/F_m_’ [49]. *qp* reflects the level of open PSII centers and it gives higher values when the photochemistry potential is high. In contrast, NPQ increases as the fluorescence is quenched due to processes other than photochemistry [50,51].

**Q_A_^−^ re-oxidation kinetics:** The Q_A_^−^ re-oxidation kinetics were recorded using the Fluorometer FL 6000F (PSI Instruments, Czech Republic) after a single turnover flash, with actinic flash duration of 50 μs and intensity of 2500 μmol photons/(m^2^ s). The total duration of the test was 50 s. The kinetic data were recorded with six points per decay. The Q_A_^−^ re-oxidation process consists of three phases (fast, medium, slow). In order to calculate their half-times of decay, the kinetics were fitted to the following exponential function [52]:

F(*t*) = F_r_ + A_1_*e^−K1*t^ + A_2_*e^−K2*t^ + A_3_*e^−K3*t^

where F(*t*) is the fluorescence at time *t*, K1, K2, and K3 are the decay rate constants, A_1_, A_2_, and A_3_ are the amplitudes of the fluorescence associated relaxation phases (fast, medium, and slow, respectively), and F_r_ is the remaining fluorescence at the end of the decay. DCMU (3-(3,4-dichlorophenyl)-1,1-dimethylurea) was also used (50 μM) as an electron transport inhibitor.

**Determination of the inactive PSII reaction centers and Q_B_ non-reducing RCs:** The oxygen evolving complex (OEC) generates oxygen after a series of oxidations of four intermediate states (S_0_→S_4_). The PSII RCs of dark-adapted cells are in the S_0_ and S_1_ states (S_0_/S_1_; slash indicates a mixture of S_x_/S_y_). In the S-states test, the five S-states are advanced stepwise by short actinic light flashes, i.e., first flash advances S_1_/S_2_, second flash S_2_/S_3_, third flash S_3_/S_4_ →S_0_ and the fourth flash S_4_ → S_0_/S_1_ [53,54]. S-states were advanced by 50 μs long flashes at 200 ms periods. S-states tests were employed for the determination of the contribution of inactive PSII RCs, because the fluorescence decay after the fourth flash (S_4_ → S_0_/S_1_) is controlled by inactive RCs. Inactive PSII were calculated according to [55]:

Inactive PSII RCs (%) = 100*ΔF_4_/[(F^3d^/F_0_) − 1]

where F^3d^ is the first fluorescence signal recorded after the third flash, ΔF_4_ = (F^4th^_99ms_/F_0_) − 1, and F^4th^_99ms_ is the fluorescence 99 ms after the fourth flash. S-states were recorded on the Fluorometer FL 6000F. For the determination of reducing site heterogeneity, a double pulse OJIP was performed in order to determine the contribution of Q_B_ non-reducing centers. More specifically two fluorescence transients were induced by two subsequent light pulses (each of 1 s duration). The Q_B_ non-reducing centers were determined as:

N_QB_ = [(F_v_/F_m_)^first pulse^ − (F_v_/F_m_)^second pulse^]/(F_v_/F_m_)^first pulse^

This section is not mandatory but can be added to the manuscript if the discussion is unusually long or complex.

## 4. Conclusions

The results of the present study revealed that *A. protothecoides* growth under mixotrophic conditions is not as favored as it is for several other microalgae. *A. protothecoides* displayed better growth rates under heterotrophy (dark conditions) probably due to some kind of photoinhibition, as NPQ analyses suggested. White and red were the best light wavelengths, in which *A. protothecoides* had the highest growth rates under mixotrophic conditions. The availability of nitrogen had the strongest effect on the biochemical composition; indeed, under nitrogen replete conditions there was a trend for protein accumulation, whereas under deplete conditions biomass tend to accumulate carbohydrates. Pigment content was affected mainly by the trophic conditions, because in the presence of glycerol (mixotrophy) pigment content was significantly decreased compared to the photoautotrophic. The light wavelength did not have any clear effect on the biomass production and the microalgal metabolites synthesis in *A. protothecoides*. The overall results suggest that trophic conditions and, in particular, the availability of nitrogen (peptone concentration), had a stronger effect on the growth, the production of metabolites, and the photosynthetic machinery than the different light wavelengths. Chlorophyll fluorescence studies suggest that the photosystem II performance and the heterogeneity of the PSII reaction centers were affected by the trophic conditions and the high peptone concentrations. The present study highlights several points that need more research, so that the overall process is better understood and optimized.

## Figures and Tables

**Table 1:** Selected calculated parameters of the OJIP and non-photochemical quenching (NPQ) tests of *A. protothecoides* cultivated under white light. Inset figures: OJIP (**a**) and NPQ (**b**) signals of the associated cultures. Data represent the average ± SD of three replicates (n = 3). (M_H_ and M_L_ stand for mixotrophy with high and low peptone, respectively, and P_H_ and P_L_ stand for photoautotrophy with high and low peptone, respectively).

**Parameter**	**Culture**				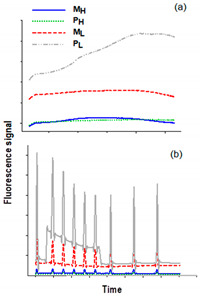
	**M_H_**	**M_L_**	**P_H_**	**P_L_**	
**ABS/RC**	54.37 ± 4.59	26.56 ± 3.7	167.23 ± 22.81	1.28 ± 0.11	
**DI_o_/RC**	50.42 ± 3.46	24.60 ± 4.72	160.94 ± 33.47	0.40 ± 0.02	
***φ*_Do_**	0.93 ± 0.09	0.91 ± 0.07	0.96 ± 0.08	0.31 ± 0.03	
***PI_ABS_***	0.01 ± 0.001	0.01 ± 0.001	0.01 ± 0.001	4.50 ± 0.48	
***NPQ***	0.04 ± 0.002	0.417 ± 0.031	0.097 ± 0.009	0.517 ± 0.027	
***qp***	0.01 ± 0.001	0.107 ± 0.005	0.01 ± 0.001	0.783 ± 0.081	

**Table 2:** Q_A_^−^ re-oxidation kinetics of *A. protothecoides* cultivated under white light. Inset figures: Q_A_^−^ re-oxidation kinetics without (**a**) and with 50 μM 3-(3,4-dichlorophenyl)-1,1-dimethylurea (DCMU) (**b**). Data represent the average ± SD of three replicates (n = 3). (M_H_ and M_L_ stands for mixotrophy with high and low peptone, respectively, and P_H_ and P_L_ stands for photoautotrophy with high and low peptone, respectively).

**Kinetic Phases**	**M_H_**	**M_L_**	**P_H_**	**P_L_**	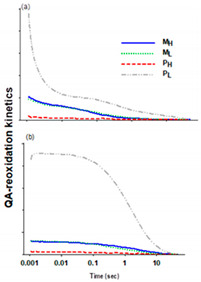
Without DCMU					
Fast Phase (*t*_1/2_, ms)	116 ± 15	207 ± 56	237 ± 16	10.3 ± 0.3	
Medium Phase (*t*_1/2_, s)	11.3 ± 0.47	20.1 ± 2.7	46.5 ± 12.4	10.1 ± 0.8	
Slow Phase (*t*_1/2_, s)	12,172 ± 1192	Not computable	18,783 ± 1691	1685 ± 190	
With 50 μM DCMU					
Fast Phase (*t*_1/2_, ms)	849 ± 61	4801 ± 3269	763 ± 56	22,847 ± 1438	
Medium Phase (*t*_1/2_, s)	43 ± 4.5	34.5 ± 0.5	46.4 ± 4.3	8.2 ± 0.9	
Slow Phase (*t*_1/2_, s)	2001 ± 304	3698 ± 557	9677 ± 1056	328 ± 25	

**Table 3:** Inactive PSII RCs and Q_B_ Non-Reducing RCs of *A. protothecoides* cultivated under white light. (M_H_ and M_L_ stands for mixotrophy with high and low peptone, respectively, and P_H_ and P_L_ stands for photoautotrophy with high and low peptone, respectively).

Culture	Inactive PSII RCs	Q_B_ Non-Reducing RCs
M_H_	Negative value	21.36% ± 3.35%
M_L_	Negative value	22.61% ± 0.93%
P_H_	Negative value	23.24% ± 5.3%
P_L_	8.5% ± 1.3%	11.10% ± 1.34%

**Table 4:** Basic experimental design of *A. protothecoides* grown mixotrophically or photoauthotrophically under different light qualities. (M_H_ and M_L_ stands for mixotrophy with high and low peptone, respectively and P_H_ and P_L_ stands for photoautotrophy with high and low peptone, respectively).

Culture Name	Glycerol (g/L)	KHCO_3_ (g/L)	Peptone (g/L)
M_H_	10	-	4
M_L_	10	-	0.5
P_H_	0	2.5	4
P_L_	0	2.5	0.5

**Table 5:** Parameters, formulae, and terms used in the OJIP test (adapted from [39]).

Parameters	Formulae	Terms
V_J_	(F_2ms_ − F_0_)/(F_m_ − F_0_)	Variable fluorescence at the J step
V_I_	(F_60ms_ − F_0_)/(F_m_ − F_0_)	Variable fluorescence at the I step
M_0_	4*(F_300μs_ − F_0_)/(F_m_ − F_0_)	Approximated initial slope of the fluorescence transients
φ_Po_	TR_o_/ABS = 1 − (F_0_/F_m_) = F_v_/F_m_	Maximum quantum yield for primary photochemistry (at *t* = 0)
φ_Do_	= 1 − Φ_Po-_(F_0_/F_m_)	Quantum yield of energy dissipation (at *t* = 0)
PI_ABS_	(RC/ABS) [φ_Po_/(1 φ_Po_)] . [Ψ_o_/(1 − Ψ_o_)]	Performance index
ABS/RC	M_0_*(1/V_J_)*(1/φ_Po_)	Absorption flux per RC

## Data Availability

The data presented in this study are available in the article.
